# SMG1 Identified as a Regulator of Parkinson’s Disease-Associated alpha-Synuclein through siRNA Screening

**DOI:** 10.1371/journal.pone.0077711

**Published:** 2013-10-30

**Authors:** Adrienne Henderson-Smith, Donald Chow, Bessie Meechoovet, Meraj Aziz, Sandra A. Jacobson, Holly A. Shill, Marwan N. Sabbagh, John N. Caviness, Charles H. Adler, Erika D. Driver-Dunckley, Thomas G. Beach, Hongwei Yin, Travis Dunckley

**Affiliations:** 1 Neurogenomics Division, Translational Genomics Research Institute, Phoenix, Arizona, United States of America; 2 Cancer and Cell Biology Division, Translational Genomics Research Institute, Phoenix, Arizona, United States of America; 3 Banner Sun Health Research Institute, Sun City, Arizona, United States of America; 4 Department of Neurology, Movement Disorders Division, Mayo Clinic Scottsdale, Scottsdale, Arizona, United States of America; University of S. Florida College of Medicine, United States of America

## Abstract

Synucleinopathies are a broad class of neurodegenerative disorders characterized by the presence of intracellular protein aggregates containing α-synuclein protein. The aggregated α-synuclein protein is hyperphosphorylated on serine 129 (S129) compared to the unaggregated form of the protein. While the precise functional consequences of S129 hyperphosphorylation are still being clarified, numerous *in vitro* and *in vivo* studies suggest that S129 phosphorylation is an early event in α-synuclein dysfunction and aggregation. Identifying the kinases and phosphatases that regulate this critical phosphorylation event may ultimately prove beneficial by allowing pharmacological mitigation of synuclein dysfunction and toxicity in Parkinson’s disease and other synucleinopathies. We report here the development of a high-content, fluorescence-based assay to quantitate levels of total and S129 phosphorylated α-synuclein protein. We have applied this assay to conduct high-throughput loss-of-function screens with siRNA libraries targeting 711 known and predicted human kinases and 206 phosphatases. Specifically, knockdown of the phosphatidylinositol 3-kinase related kinase SMG1 resulted in significant increases in the expression of pS129 phosphorylated α-synuclein (p-syn). Moreover, SMG1 protein levels were significantly reduced in brain regions with high p-syn levels in both dementia with Lewy bodies (DLB) and Parkinson’s disease with dementia (PDD). These findings suggest that SMG1 may play an important role in increased α-synuclein pathology during the course of PDD, DLB, and possibly other synucleinopathies.

## Introduction

The α-synuclein (SNCA) protein is intricately involved in the pathogenesis of Parkinson’s disease and other synucleinopathies, including dementia with Lewy bodies (DLB) and multiple system atrophy (MSA). Each disorder is characterized by protein aggregates containing α-synuclein protein that has been hyperphosphorylated on serine 129 (p-Syn). In PD and DLB p-Syn is found in intraneuronal inclusions called Lewy bodies and in Lewy neurites. In MSA p-Syn is found in neurons as well as glial cytoplasmic inclusions in oligodendrocytes [1]. The importance of p-Syn in the pathogenesis of synucleinopathies is further strengthened by genetic findings showing that point mutations in the SNCA gene [2], [3], [4] and SNCA locus duplication and triplication [5], [6], [7] cause autosomal dominant forms of PD that manifest with neuropathological features in common with sporadic PD, including the presence of Lewy bodies with p-Syn aggregates.

*In vitro*, α-synuclein protein undergoes both serine and tyrosine phosphorylations on Ser87, Ser129, Tyr125, Tyr133, and Tyr136 [8], [9], [10], [11]. However, only Ser87, Ser129, and Tyr125 have been observed to be phosphorylated *in vivo*. Of these, phosphorylation at Ser129 has been seen in pre-Lewy body stages of disease and appears to progress with disease [12]. In addition, careful testing of neuropathologically staged PD samples has shown that Ser129 phosphorylation precedes the presentation of overt Lewy pathology [13]. These combined findings indicate that serine 129 phosphorylation is likely to be an early event in disease pathology. Identifying the kinases and phosphatases responsible for regulating this phosphorylation site may hold some promise for therapeutic development to alter the course of synuclein pathology in PD and other synucleinopathies.

Numerous kinases have been linked to Ser129 phosphorylation in various *in vitro*, cell culture, and *in vivo* models. These include most prominently the polo-like kinases (PLKs) 1, 2, and 3 [14], [15], [16] and G-protein receptor coupled kinases 1, 2, 5, and 6 [9].Protein phosphatases have been less extensively studied. Results thus far suggest that protein phosphatase 2A (PP2A), which has a broad range of substrates and specificities, can dephosphorylate Ser129 of α-synuclein [17]. These studies have provided valuable information regarding the phosphorylation of α-synuclein. But the numerous kinases implicated to date suggest that there may be functionally redundant signaling pathways converging to regulate this phosphorylation and, further, that there may be additional kinases and phosphatases involved that have yet to be described.

To identify additional kinases and phosphatases that regulate p-Syn levels, we sought first to develop a high-content, high-throughput cellular assay to accurately measure the levels of pS129 (p-Syn) and total α-synuclein (t-Syn) protein. We then leveraged this assay to screen kinase and phosphatase siRNA libraries to identify those kinases and phosphatases with the most robust effects on p-Syn and t-Syn levels. Results implicate several additional kinases and phosphatases in Ser129 phosphorylation. We show that knockdown of one of these kinases, the phosphatidylinositol 3-kinase related kinase SMG1, significantly enhances expression of both p-Syn and t-Syn levels and, further, that expression of this kinase is significantly reduced in brain samples from neuropathologically confirmed cases of Parkinson’s disease with dementia (PDD) and dementia with Lewy bodies (DLB) in brain regions known to have significantly elevated p-Syn. These results suggest that reduced SMG1 expression may be a contributor to α-synuclein pathology in these diseases.

## Materials and Methods

### Inducible Expression of α-synuclein in Cell Culture

The 3D5 cells are a neuroblastoma-derived cell line transfected with the pTetOff-Neo vector (Clontech) expressing full-length, wild-type human α-synuclein and were obtained from the Shu-Hui lab (Department of Neuroscience, Mayo Clinic College of Medicine, Jacksonville, FL) [18]. The cells were maintained in selection growth media consisting of Dulbecco’s Modified Eagle Medium (DMEM)/10% fetal bovine serum supplemented with 400 µg/mL Geneticin, 1 µg/mL Puromycin and 2 µg/mL Tetracycline.

Prior to experiments, the 3D5 cells were conditioned in medium without Tetracycline for 3 days prior to transfection to initiate α-synuclein expression using the Tet-Off system. After conditioning, the cells were harvested, transfected with siRNAs and were continued to be grown in tetracycline deficient media for an additional 96 hours. Overall, the 3D5 cells were induced for α-synuclein expression for 7 days.

### RNAi Screening

The 3D5 cells were reverse-transfected with the Validated Kinase (version 4) siRNA library covering 711 genes and the Phosphatase library covering 206 genes (Qiagen). Four oligomers were tested per gene. Two microliters of 0.667 mM individual siRNA were printed into 96-well black, clear-bottomed plates treated with poly-D-lysine (Greiner) using a Biomek FX Laboratory Automation Workstation (Beckman Coulter). The transfection reagent Lipofectamine 2000 (Invitrogen, Carlsbad, CA) was used at a concentration of 150 nL/well. Non-targeting/scrambled negative siRNA controls (GFP, AllStar-NonSilencing (ASNS), and NonSilencing (NS)) as well as the positive transfection control (Allstar Cell Death Control (ACDC)) from Qiagen were also used in the screen. All siRNA sequences were screened at a final concentration of 13 nM. Following transfection the cells were incubated for an additional 96 hours in the absence of tetracycline before the cells were fixed and stained for levels of total and phosphorylated SNCA.

### Antibodies and Dyes for Immunofluorescence

A rabbit polyclonal antibody to human α-synuclein (Stressgen) and a mouse monoclonal antibody to serine 129 phosphorylated human α-synuclein (Wako Pure Chemical Industries, Ltd) were used as primary antibodies. FITC conjugated goat anti-rabbit IgG and Cy5 conjugated goat anti-mouse IgG antibodies (Jackson ImmunoResearch) were used as secondary antibodies for the fluorescent detection of total and phosphorylated α-synuclein expression levels. Hoechst 33342 dye (Invitrogen) was used to stain the nucleus of the cells.

### Immunofluorescence Staining

Cells in the microplates were fixed using 4% Paraformaldehyde (PFA) for 15 min at room temperature. Following fixation, the cells were treated with 0.1% Triton X-100 in PBS 5 minutes to permeabilize the nuclear component. The cells were blocked in 4% Goat Serum, 0.05% Tween-20, 0.02% Sodium Azide in Tris-buffered saline solution (TBS, pH 7.6). Blocking solution was also used to prepare all primary and secondary antibody solutions. The human anti-SNCA antibody was probed at a dilution of 1∶500 for 3 hours at room temperature. The anti-pS129 SNCA antibody was probed at a dilution of 1∶1000 for 3 hours at room temperature. The FITC conjugated goat anti-rabbit secondary antibody solution was probed at a dilution of 1∶300 for 1 hour at room temperature. The Cy5 conjugated goat anti-mouse secondary antibody solution was probed at a dilution of 1∶500 for 1 hour at room temperature. The Hoechst 33342 nuclear dye was also used at a concentration of 1 µg/mL and was added to each secondary antibody solution. After application of both primary and secondary antibody solutions, the cells were washed 3 times for 5 minutes with a 100 mM NaCl, 0.05% Tween-20 TBS solution prior to imaging.

### High-content Fluorescence Imaging

After the cells were probed for t-syn, p-syn, and nuclear DNA content, fluorescent images were obtained from each well using the ImageXpress Micro (Molecular Devices). Three fluorescent measurements were obtained: Hoechst dye (Blue stain measuring nuclear DNA) used to quantify the number of cells per well; FITC (green) conjugated secondary antibody measuring t-syn; Cy5 (red) conjugated secondary antibody measuring p-syn. On average, over 100 cells were acquired for each field imaging. From the intensities of t-syn (FITC, green) and p-syn (Cy5, red), a cellular average for each channel was calculated using the number of cells. These fluorescent averages of p-syn/t-syn were then used to generate a ratio.

### Immunostaining Analysis

The ratio of p-syn/t-syn was used as an input for data analysis using CellHTS2 software (http://web-cellhts2.dkfz.de/cellHTS-java/cellHTS2/) [19]. In this analysis, the data was first normalized by B score method and the variance adjusted per plate [20]. B score corrects for spatial effects within the plate, such as gradients and edge effects. The variance adjustment per plate was applied to account for the plate-to-plate variation in the cell line screen. The data was then standardized by a robust Z-score method using plate median and median absolute deviation (MAD) [19], where Z = 0 represents no effect on changes between the ratio of p-syn/t-syn. Positive Z-scores represent increases in the ratio of p-syn/t-syn while negative Z-scores represent decreases in this ratio. In our screen, Z-score data approximated a normal distribution, thereby allowing us to choose Z >2 and Z<−2 standard deviations from the median as the siRNA sequences that significantly increase the ratio.

### Western Analysis

Following transfection with siRNAs (see above), on ice, media was aspirated off and cells washed with PBS. Cold lysis buffer (0.8–1 ml) (Complete Lysis-M buffer (Roche Applied Science, 04719964001)+protease inhibitor tablet (provided with buffer)+phosphatase inhibitor cocktails 2 (P5726, Sigma) & 3 (P0044, Sigma) was added directly to wells and left on ice briefly then scraped with a rubber policeman and transferred to a microfuge tube. Tube was placed on ice and agitated periodically. Lysates were centrifuged at 14,000 g for 15 min at 4°C to remove cell debris. Protein concentration was determined using the BCA protein assay (Pierce). Protein from lysates (20 µg) was separated by SDS-PAGE (3–8% Tris-Acetate and 4–12% Bis-Tris) and transferred to nitrocellulose. Membrane was blocked in 5% BSA for one hour at RT. Membranes were probed with primary antibody overnight at 4°C on a rocker. Membranes were subsequently washed with TBS-T and probed with secondary antibody for forty-five minutes. Membranes were further washed and developed with Super Signal West Femto Maximum Sensitivity Substrate Kit (Promega) and imaged. To test multiple primary antibodies, membranes were stripped for 15 minutes at RT using ReBlot Plus Mild Antibody Stripping Solution (Millipore). Membranes were then washed again for 5 minutes at RT then blocked for one hour in 5% BSA. Membrane was re-probed overnight at 4°C with an anti-Tubulin antibody. Antibodies and dilutions used were: α-SMG1 (1∶1,500, Abcam, ab80600); α-SNCA (1∶2,000, Abcam, ab27766); α-pS129 SNCA (1∶750, Abcam, ab59264); α-UPF1 (1∶1,000, Abcam, ab10510); α-Tubulin (1∶1,000, Cell Signaling, 2148), α-TIA1 (1∶1,000, Santa Cruz, 1751); (Secondary antibodies: Rabbit Anti-Goat, 1∶90,000, Sigma A5420; Goat Anti-Mouse, 1∶25,000, Jackson Immuno 115-035-071; Goat Anti-Rabbit, 1∶25,000, Jackson Immuno 111-035-144). The SMG1 siRNA sequences were: Sequence A, ATCGATGTTGCCAGACTACTA; Sequence B, CACCATGGTATTACAGGTTCA; Sequence C, ATGGGTCAGGTTCGAAGTCAA; Sequence D, TTCGTTTGCATCACAGTTTAA.

For determination of levels of SMG1 protein in the brain, fresh frozen cryostat sections were removed from slides with a razor blade over dry ice and placed in a cold 1.5 ml microfuge tube. Cold lysis buffer (Complete Lysis-M buffer (Roche Applied Science, 04719964001) + protease inhibitor tablet (provided with buffer)+phosphatase inhibitor cocktails 2 (P5726, Sigma) & 3 (P0044, Sigma)) was added (0.8–1 ml) and the tube was placed on wet ice and agitated periodically. Lysates were centrifuged at 14,000 g for 15 min at 4°C to remove cell debris. Protein was quantitated as above and run on westerns as described above.

## Results

### High-Content α-Synuclein Phosphorylation Assay

**Figure 1:**
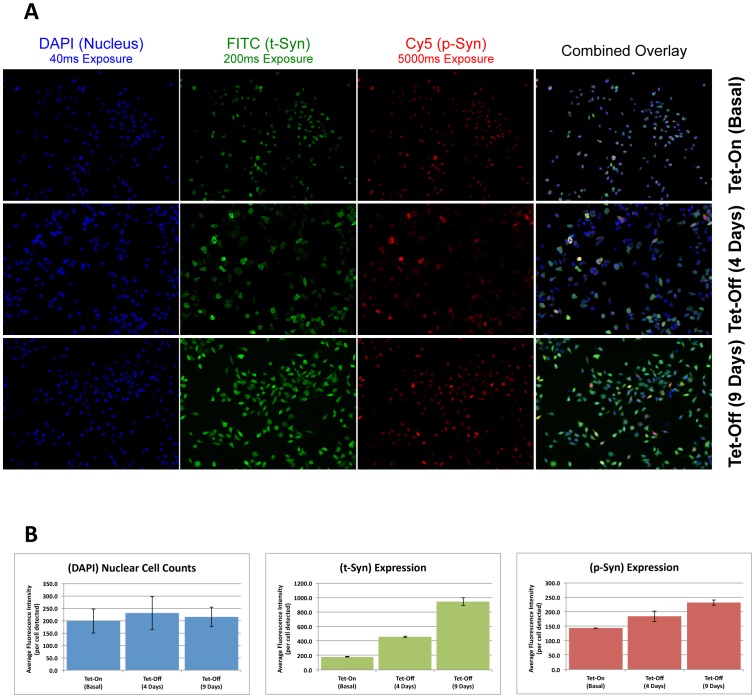
Sensitive fluorescence detection of p-syn and t-syn over time. Shown in A are fluorescence images of nuclei (Blue), t-syn (green), and p-syn (red) with the exposure time of 40 ms, 200 ms, and 5000 ms, respectively. Cells were imaged (top row) and subsequently removed from tetracycline to induce α-synuclein expression. Cells were then reimaged at day 4 (middle row) and day 9 (bottom row). Shown in B, image intensity increased following induction of the synuclein transgene, consistent with increased p-syn and t-syn expression over time.

To facilitate the identification of kinases and phosphatases that may be important in PD relevant pathologic phosphorylation of α-synuclein protein, we developed a cell-based, high-throughput immunofluorescence assay for the rapid detection and quantitation of both total α-synuclein (t-syn) and pS129 α-synuclein (p-syn). Due to the fact that constitutive, high level expression of α-synuclein was toxic to host neuroblastoma cells (data not shown), we opted for an inducible system. The assay uses the BE(2)-M17D human neuroblastoma cell line expressing full-length α-synuclein protein under the control of the tet-off promoter (3D5 cells described in Takahashi et al. [18]). To minimize the assay variation, we induced a large flask of cells simultaneously before seeding them in individual 96-wells to optimize consistency in α-synuclein expression. Published data with this inducible cell line demonstrates that t-syn and p-syn levels rise steadily from day 1 post-induction to beyond day 7 of induction. To demonstrate that our fluorescence assay reflects increased α-synuclein expression, we performed immunostaining at varying days following induction of the Tet-off promoter (Figure 1). The expression of α-synuclein was induced by removing tetracycline from growth media and t-syn and p-syn levels were assessed at day zero (Figure 1A, top row), following 4 days (Figure 1A, middle row) and 9 days (Figure 1A, bottom row) of induction. All of the various induction conditions were imaged at a fixed exposure time for each fluorescence channel. With the understanding that the number of cells imaged may likely vary, the average fluorescence intensity per cell was quantified for each respective channel. With the average intensity of nuclear staining remaining stable, the intensity of t-syn and p-syn both increased significantly following 4 days and 9 days of induction of the Tet-off promoter, at the rate of >200% and >25% for t-syn and p-syn at each time point (Figure 1B). Comparatively, these increased average fluorescence intensity values demonstrate the increased expression of t-syn and p-syn and coincides with the visual images (Figure 1A) compared to the Tet-On negative control (top panels).

### siRNA Library Screening

In order to identify genes that affect t-syn and p-syn levels, we screened siRNA libraries targeting 711 known and predicted kinases and 206 phosphatases. Each kinase and phosphatase was targeted with four unique siRNA sequences. Following data acquisition of the screen, transfection efficiency was assessed to ensure effectiveness of the siRNA knockdown. Comparison of the average number of cells detected when treated with either non-targeting or cytotoxic control siRNA was used for this metric. An overall transfection efficiency of >85% was achieved throughout the screen (data not shown), providing confidence that any observed changes in the screen likely resulted from siRNA activity.

The sample data was then submitted for p-syn/t-syn ratio calculation and analysis using CellHTS2 software. The ratio of the cellular fluorescence averages of p-syn and t-syn was used to generate a robust Z-score. This Z-score data approximated a normal distribution and a stringent threshold of Z<−2 and Z >2 was selected, resulting in 317 and 313 siRNA respectively, as hits. In a normal distribution at Z<−2 and Z >2, the two-tailed probability α = 0.0455, which implies that the probability of a siRNA giving the observed effect by chance is <0.0455. In order to reduce the number of false positives from the hits obtained by the Z-score method described above, we additionally filtered the list for a percent increase or decrease in the p-syn/t-syn ratio of 20% for each siRNA compared to the average ratio seen with non-targeting siRNA controls on each plate. This trimmed the list of hits to 36 genes at Z<−2 (See Table 1) and 27 genes at Z >2 (See Table 2). The siRNA screening data for the full kinase and phosphatase siRNA libraries are summarized in Table S1. Importantly, even though we have used a “validated” Qiagen siRNA library, we cannot rule out the possibility that any negative results observed in the screening data may result from incomplete knockdown of the intended target gene.

**Table 1:** Kinases and Phosphatases that decrease the ratio of p-syn/t-syn.

				FoldChange(Zscore)			
Gene	Entrez ID	Description	siCount	siRNA1	siRNA2	siRNA3	siRNA4
PLK1	5347	Polo-like kinase 1 (Drosophila)	4	0.65 (−5.43)	0.55 (−10.22)	0.53 (−10.57)	0.52 (−5.89)
SPHK1	8877	Sphingosine kinase 1	3	0.61 (−5.10)	0.65 (−3.28)	0.77 (−4.46)	
MVK	4598	Mevalonate kinase (mevalonic aciduria)	2	0.79 (−3.33)	0.70 (−6.95)		
PHKA1	5255	Phosphorylase kinase, alpha 1 (muscle)	2	0.73 (−3.99)	0.70 (−5.59)		
HK1	3098	Hexokinase 1	2	0.74 (−2.04)	0.77 (−6.13)		
LMTK2	22853	Lemur tyrosine kinase 2	2	0.67 (−2.08)	0.79 (−2.64)		
TNIK	23043	TRAF2 and NCK interacting kinase	2	0.78 (−2.16)	0.79 (−2.20)		
CDK5R2	8941	Cyclin-dependent kinase 5, regulatory subunit 2 (p39)	2	0.76 (−2.45)	0.79 (−2.75)		
PGK1	5230	Phosphoglycerate kinase 1	2	0.79 (−3.07)	0.77 (−3.44)		
PPP2R1A	5518	Protein phosphatase 2 (formerly 2A), regulatory subunit A,alpha isoform	2	0.79 (−3.91)	0.54 (−4.90)		
PTPRA	5786	Protein tyrosine phosphatase, receptor type, A	2	0.79 (−5.44)	0.67 (−5.00)		
TSSK1B	83942	Testis-specific serine kinase 1B	2	0.69 (−3.11)	0.78 (−3.73)		
CDKL1	8814	Cyclin-dependent kinase-like 1 (CDC2-related kinase)	1	0.69 (−2.25)			
ACP1	52	Acid phosphatase 1, soluble	1	0.63 (−4.10)			
CDK3	1018	Cyclin-dependent kinase 3	1	0.79 (−2.43)			
MERTK	10461	C-mer proto-oncogene tyrosine kinase	1	0.62 (−2.24)			
MAGI2	9863	Membrane associated guanylate kinase, WW and PDZdomain containing 2	1	0.76 (−2.36)			
NEK9	91754	NIMA (never in mitosis gene a)- related kinase 9	1	0.79 (−2.01)			
CKB	1152	Creatine kinase, brain	1	0.73 (−9.76)			
PLK2	10769	Polo-like kinase 2 (Drosophila)	1	0.73 (−2.03)			
SPHKAP	80309	SPHK1 interactor, AKAP domain containing	1	0.72 (−2.46)			
PTPRB	5787	Protein tyrosine phosphatase, receptor type, B	1	0.61 (−5.69)			
PPP1R11	6992	Protein phosphatase 1, regulatory (inhibitor) subunit 11	1	0.73 (−2.10)			
PTPDC1	138639	Protein tyrosine phosphatase domain containing 1	1	0.77 (−2.49)			
PPP2R2B	5521	Protein phosphatase 2 (formerly 2A), regulatory subunit B,beta isoform	1	0.74 (−2.20)			
PIK3R1	5295	Phosphoinositide-3-kinase, regulatory subunit 1 (p85 alpha)	1	0.79 (−7.20)			
WEE1	7465	WEE1 homolog (S. pombe)	1	0.70 (−5.46)			
PSPH	5723	Phosphoserine phosphatase	1	0.79 (−7.31)			
PRKAB2	5565	Protein kinase, AMP-activated, beta 2 non-catalytic subunit	1	0.77 (−4.33)			
LIMK2	3985	LIM domain kinase 2	1	0.71 (−2.70)			
SRPK1	6732	SFRS protein kinase 1	1	0.74 (−4.36)			
PPP2R2D	55844	Protein phosphatase 2, regulatory subunit B, delta isoform	1	0.77 (−3.00)			
EPHA1	2041	EPH receptor A1	1	0.79 (−3.98)			
STK19	8859	Serine/threonine kinase 19	1	0.72 (−3.65)			
ITPK1	3705	Inositol 1,3,4-triphosphate 5/6 kinase	1	0.60 (−7.31)			
PPP2R2A	5520	Protein phosphatase 2 (formerly 2A), regulatory subunit B,alpha isoform	1	0.73 (−3.65)			

Data were standardized using the B-score method as described in the text and subsequently filtered for Z-scores<−2. The average fold change in the ratio of p-syn/t-syn (relative to the ratio seen in the NS siRNA control samples) for the multiple significant siRNAs at the Z<−2 cut-off are indicated in the column next to the gene symbols. The number of siRNAs showing significance at each Z score cut-off are indicated.

**Table 2:** Kinases and Phosphatases that increase the ratio of p-syn/t-syn.

				FoldChange (Zscore)			
Gene	Entrez ID	Description	siCount	siRNA1	siRNA2	siRNA3	siRNA4
SMG1	23049	PI-3-kinase-related kinase SMG-1	4	2.17 (17.86)	1.76 (11.00)	4.96 (42.87)	1.40 (2.47)
DSTYK	25778	dual serine/threonine and tyrosine protein kinase	2	1.24 (2.07)	3.1 (35.81)		
DUSP7	1849	Dual specificity phosphatase 7	2	1.23 (4.65)	1.26 (2.43)		
MARK1	4139	MAP/microtubule affinity-regulating kinase 1	2	1.28 (3.16)	1.19 (2.65)		
PAK3	5063	P21 (CDKN1A)-activated kinase 3	2	1.41 (5.76)	1.42 (3.27)		
PLK3	1263	Polo-like kinase 3 (Drosophila)	2	1.31 (3.81)	1.41 (3.11)		
PPP3R1	5534	Protein phosphatase 3 (formerly 2B), regulatory subunit B,alpha isoform	2	1.64 (16.51)	1.32 (2.23)		
SIK2	23235	salt-inducible kinase 2	2	1.22 (6.31)	1.50 (4.84)		
SNRK	54861	SNF related kinase	2	1.66 (8.97)	1.38 (2.99)		
TGFBR2	7048	Transforming growth factor, beta receptor II (70/80 kDa)	2	1.76 (12.6)	1.44 (3.50)		
TPK1	27010	Thiamin pyrophosphokinase 1	2	1.21 (3.54)	1.24 (2.55)		
C17orf75	64149	Chromosome 17 open reading frame 75	1	1.23 (3.34)			
CHEK1	1111	CHK1 checkpoint homolog (S. pombe)	1	1.25 (3.53)			
DGKB	1607	Diacylglycerol kinase, beta 90 kDa	1	1.40 (2.02)			
DGKE	8526	Diacylglycerol kinase, epsilon 64 kDa	1	1.42 (3.43)			
DGKK	139189	Diacylglycerol kinase, kappa	1	1.68 (4.96)			
DLG1	1739	Discs, large homolog 1 (Drosophila)	1	2.37 (15.35)			
DUSP3	1845	Dual specificity phosphatase 3 (vaccinia virus phosphataseVH1-related)	1	1.27 (2.64)			
GK2	2712	Glycerol kinase 2	1	1.51 (5.48)			
GKAP1	80318	G kinase anchoring protein 1	1	1.23 (2.63)			
INPP1	3628	Inositol polyphosphate-1-phosphatase	1	1.21 (2.11)			
INPP5B	3633	Inositol polyphosphate-5-phosphatase, 75 kDa	1	1.26 (2.84)			
MAP4K5	11183	Mitogen-activated protein kinase kinase kinase kinase 5	1	1.22 (4.13)			
NEK1	4750	NIMA (never in mitosis gene a)-related kinase 1	1	1.38 (2.42)			
MTMR12/PIP3AP	54545	Myotubularin related protein 12	1	1.53 (6.29)			
PTPRN	5798	Protein tyrosine phosphatase, receptor type, N	1	1.49 (4.27)			
TXK	7294	TXK tyrosine kinase	1	2.75 (33.17)			

Data were standardized as in Table 1. The average fold change in the ratio of p-syn/t-syn (relative to the ratio seen in the NS siRNA control samples) for the multiple significant siRNAs at the Z >2 cut-off are indicated for each siRNA. The Z scores for each siRNA are in parentheses after the fold change. The number of siRNAs showing significance at each Z score cut-off are indicated.

Among the list of genes that reduced the p-syn/t-syn ratio were the polo-like kinases 1 and 2 (Table 1), which have previously been implicated in the generation of p-syn [14], [15]. Interestingly, 9 additional kinases and 2 phosphatases had two or more siRNAs that each showed significant reductions in the p-syn/t-syn ratio. The non-silencing control is set to a normalized ratio of 1.0 for comparison. While being mostly novel in their association to p-syn, the specific biological or disease relevance of these findings remains to be elucidated.

**Figure 2:**
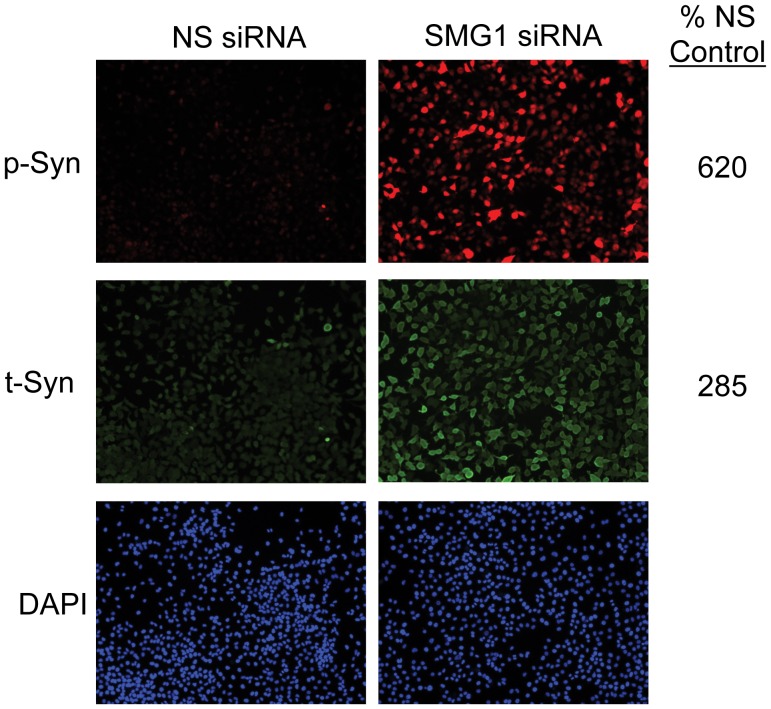
SMG1 siRNA causes a large increase in both total α-synuclein (t-syn) and pS129 phosphorylated α-synuclein (p-syn). Shown are results from the siRNA screen for one of the four significant SMG1 siRNAs. p-syn is shown in red and t-syn in green. The level of p-syn and t-syn as a percent of expression seen in control non-silencing siRNA samples is indicated. DAPI stained nuclei, which were used to calculate cell numbers, are shown in the blue panels.

A total of 9 kinases and 2 phosphatases increased the p-syn/t-syn ratio, as measured by at least two independent siRNAs (Table 2). The largest effect was seen with the PI-3-kinase-related kinase (SMG1) gene (Figure 2 and see Figure S1 for a higher magnification 20× image), for which all of the four unique siRNA sequences screened showed significant increases relative to the non-silencing control (Table 2). In fact, SMG1 silencing showed the largest effect of all genes tested.

### Validation of SMG1 Effects on α-synuclein Expression

**Figure 3:**
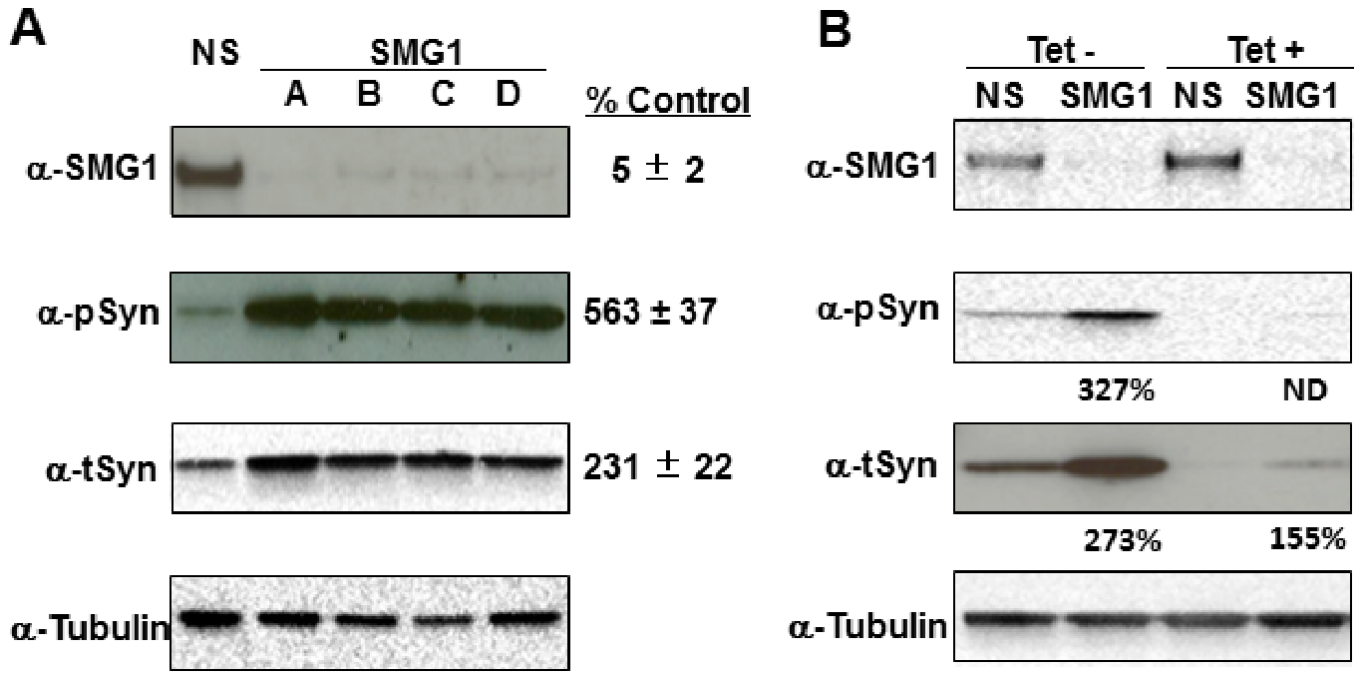
Silencing SMG1 expression increases p-syn and t-syn levels. Shown in A are westerns confirming that SMG1 silencing using four unique siRNA sequences causes large increases in p-syn and t-syn, confirming screening results shown in Figure 2. Expression levels as a % of non-silencing (NS) siRNA control are under each western. Values are the average of all four siRNA sequences. siRNAs used are indicated across the top of the westerns. Antibodies used are anti-SMG1 (α-SMG1), anti-pS129 α-synuclein (α-pSyn), anti-α-synuclein (α-tSyn), and anti-α/β Tubulin (α-Tubulin). Shown in B are westerns demonstrating that SMG1 silencing increases expression of both endogenous α-synuclein (Tet+ samples) and transgenic α-synuclein that is expressed from the tet-off promotor (Tet- samples). Expression levels as a % of NS siRNA control are indicated beneath each sample. ND stands for not detected. Antibodies used are the same as in A. Results are representative of three independent replicates.

Given the robust results with SMG1 siRNAs (Figure 2 and Table 2), we confirmed this result using secondary western analyses. Cells were re-transfected with each of the four SMG1 siRNAs individually, as was done in the discovery screen, and cell lysates were harvested for western analyses. Results confirmed that all four SMG1 siRNAs significantly reduced SMG1 protein expression, and significantly increased the levels of t-syn protein by 2.3-fold and p-syn protein by 5.6-fold (Figure 3A). This fold increase of t-syn and p-syn protein expression by knockdown with siRNA against SMG1 is similar to that detected from primary high content screening (2.8-fold and 6.2-fold in Figure 2, respectively).

We next tested if SMG1 knockdown affected the expression of endogenous t-syn or only of the t-syn expressed from the tet-off transgene in this cell line. To do this, SMG1 was knocked down in cells that were grown in medium that either contained tetracycline (Tet+) to repress transgene expression or in medium lacking tetracycline (Tet−) to induce transgene expression. Results showed that SMG1 knockdown increased t-syn levels from both the endogenous SNCA gene (Tet+ condition) and the tetracycline regulatable SNCA transgene (Tet− condition) (Figure 3B). In this experiment, we were unable to detect significant levels of p-syn for quantitation when measuring endogenous protein, indicating an extremely low baseline level of p-syn. However, levels of t-syn were significantly upregulated from the endogenous locus when SMG1 was knocked down, leading to the conclusion that loss of SMG1 increases endogenous α-syn levels, in addition to the significant increases in p-syn and t-syn observed when α-syn is induced from the Tet promoter.

### SMG1 Effects are Independent of mRNA Surveillance Activity

**Figure 4:**
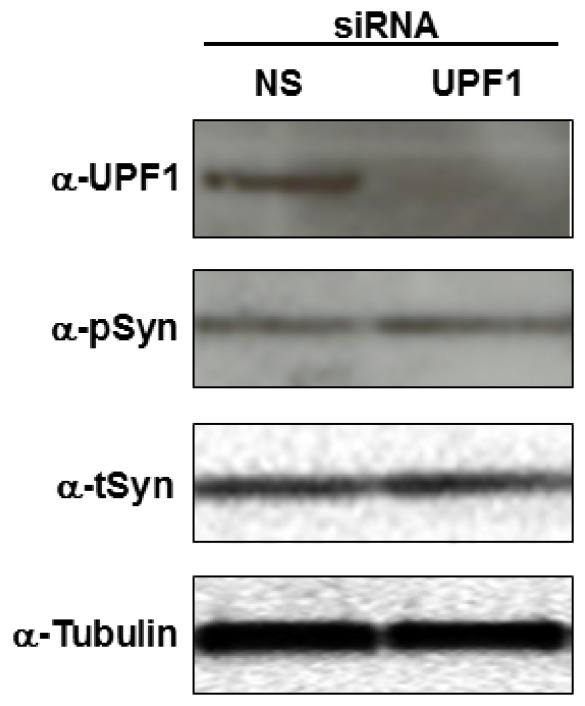
SMG1 effects on α-synuclein expression are independent of its function in the mRNA surveillance pathway. Silencing the UPF1 protein, a key regulator of mRNA surveillance, has no effect on either p-syn or t-syn levels, indicating that disruption of the mRNA surveillance pathway is not the mechanism through which SMG1 affects α-synuclein. The antibodies are the same as in Figure 3, with the addition of anti-UPF1 antibody (α-UPF1). siRNAs used are indicated across the top of the westerns. Results are representative of at least 3 independent replicates.

The SMG1 kinase is a key regulator of mRNA surveillance, a well-conserved post-transcriptional quality control mechanism that functions to degrade mRNAs containing premature translation termination codons or those mRNAs that have been incorrectly spliced. Given this well described role for SMG1, we were interested to determine if the p-syn and t-syn effects caused by loss of SMG1 resulted from reduced activity of the mRNA surveillance pathway. To do this, we used siRNA to silence expression of the *Up*stream *f*rameshift protein 1 (UPF1) gene, the key target of SMG1 activity in the mRNA surveillance pathway [21], [22], [23], [24]. As seen in Figure 4, siRNA targeting UPF1 knocked down UPF1 protein expression, yet failed to increase t-syn or p-syn expression (Figure 4). This suggests that SMG1 effects on α-syn are distinct from its mRNA surveillance activity.

### SMG1 Effects on α-synuclein are Independent of p53

**Figure 5:**
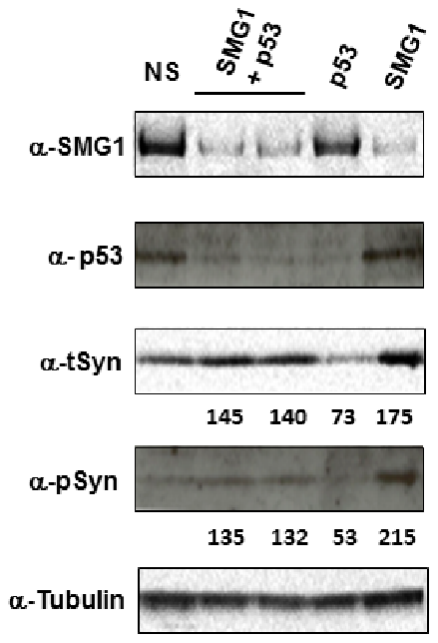
SMG1 does not function through p53 to affect α-synuclein. Shown are results wherein SMG1 and p53 were knocked down either individually or in combination to assess effects on α-synuclein. Westerns testing α-synuclein levels in these knockdown experiments show that SMG1 mediated activation of p53 is not involved in the increases in α-synuclein. Expression levels of α-synuclein for each siRNA condition normalized to the non-silencing (NS) siRNA control are shown beneath the total synuclein (α-tSyn) western. Antibodies used were as in Figure 3, with anti-p53 antibody (α-p53) included. siRNAs used are indicated across the top of the westerns, which are representative of three independent replicates.

SMG1 has been shown to phosphorylate and activate p53 protein in response to genotoxic stress [25]. While there is not a substantive reason to presume that there might be increased genotoxic stress in the α-synuclein cell model used, given that p53 can be a downstream effector of SMG1, we nevertheless tested if p53 was involved in the increase in t-syn levels following SMG1 knockdown. To do this, cells were singly transfected with siRNAs targeting either SMG1 or p53 and were also transfected with both siRNAs to simultaneously knockdown both targets. Results demonstrated that levels of t-syn are not significantly affected by p53 knockdown and, more importantly, that p53 knockdown does not prevent the increased t-syn that is observed when SMG1 expression is removed (Figure 5). We conclude that SMG1 does not function through p53 to constrain t-syn levels.

### SMG1 Effects on α-synucleininvolve TIA1 Function

SMG1 has been shown recently to be required for the formation of stress granules in the cytoplasm of cells under oxidative stress conditions [26]. The UPF1 protein also colocalizes to these stress granules, but in a form that has not undergone the SMG1-catalyzed activating phosphorylations required for mRNA surveillance activity. In those studies, SMG1 also continued to colocalize to stress granules following UPF1 depletion with siRNA. Therefore, one possibility for explaining the seemingly disparate roles of SMG1 and UPF1 in affecting α-synuclein levels (compare results in Figures 3A and 4) is that the loss of SMG1 could prevent formation of stress granules, which may function to restrict α-synuclein expression in the cell. To test this specific hypothesis, we knocked down expression of the cytotoxic granule-associated RNA binding protein (TIA1), either alone or in combination with SMG1 knockdown, and quantitated the levels of p-syn and t-syn. The TIA1 protein is an RNA binding protein involved in stress granule formation.

**Figure 6:**
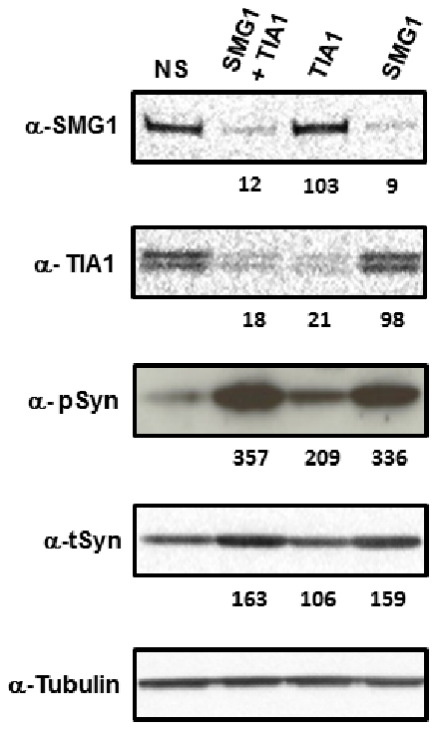
Silencing of the stress granule protein TIA1 increases p-syn. Shown are results wherein SMG1 and TIA1 were knocked down either individually or in combination to assess effects on α-synuclein. Expression levels of p-syn and t-syn for each siRNA condition normalized to the non-silencing (NS) siRNA control are shown beneath each lane on the westerns. Results are representative of at least three independent replicates. Antibodies used were as in Figure 3, with anti-TIA1 antibody (α-TIA1) included. siRNAs used are indicated across the top of the westerns.

There were several interesting results from this experiment. First, TIA1 siRNA, which reduced TIA1 expression to ∼20% of control, sharply increased p-syn levels to more than 200% of non-silencing control siRNA (Figure 6). This result, while less dramatic in magnitude, resembles the increased p-syn seen with SMG1 knockdown (Figure 6, Lane 4 and also Figure 3A). Second, the combination of SMG1 and TIA1 siRNA did not result in a synergistic increase in p-syn beyond either SMG1 or TIA1 siRNAs alone (Figure 6, compare lanes 3 and 4 to lane 2). These two findings are consistent with TIA1 and SMG1 proteins functioning in a common pathway. Third, t-syn levels were not significantly affected by TIA1 knockdown alone, suggesting that perhaps p-syn may be more sensitive to the more subtle effects of TIA1 knockdown relative to SMG1 knockdown (Figure 6 - α-tSyn panel) or to the fact that we achieved a maximum of ∼80% knockdown of TIA1, while routinely and consistently reaching more than 95% knockdown of SMG1. These combined results suggest the possibility that SMG1 could function to regulate α-synuclein levels through its recently described function in stress granule formation.

### SMG1 is Downregulated in Brain Regions with High pS129 α-synuclein Levels

**Figure 7:**
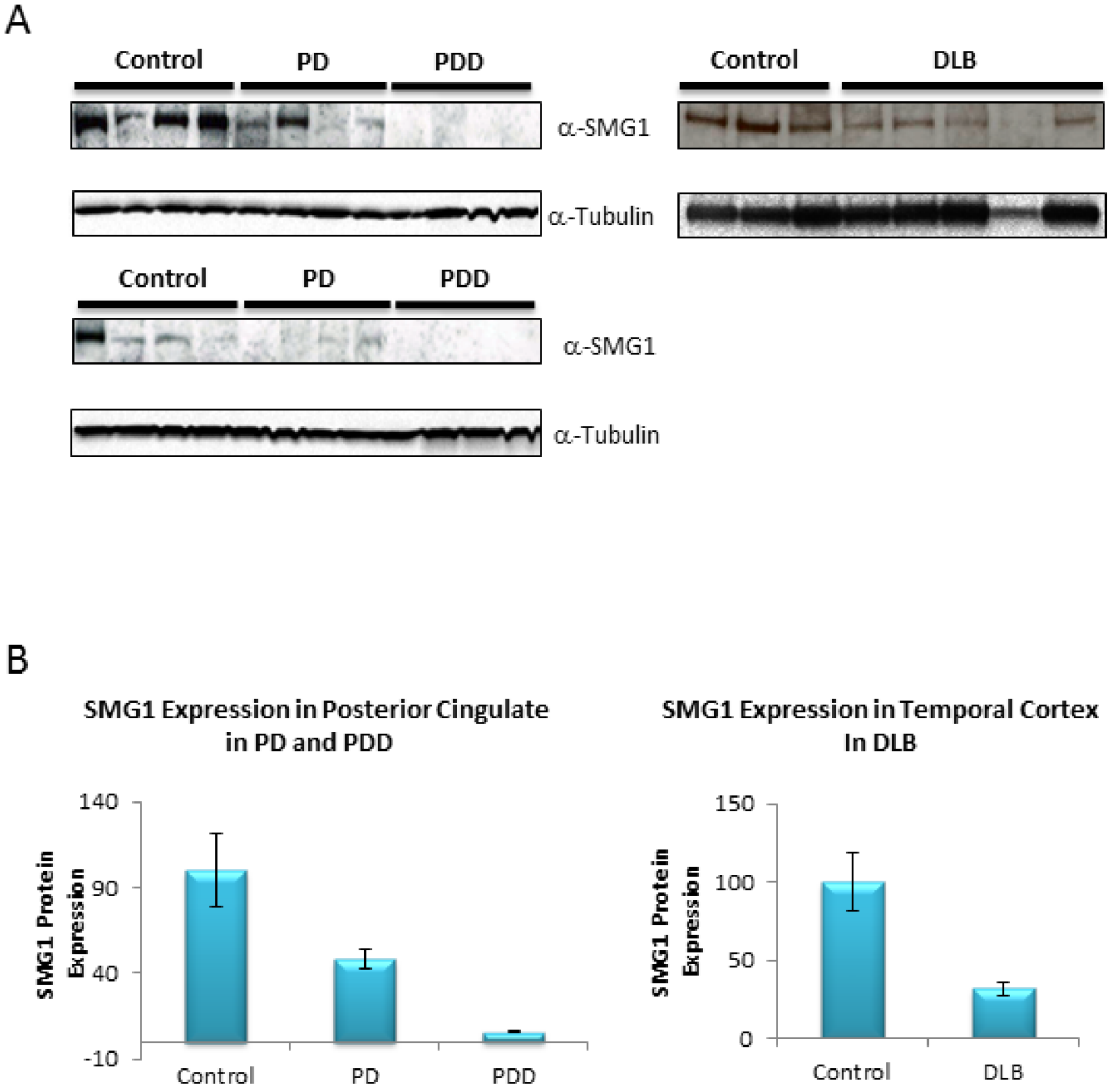
SMG1 expression is reduced in Parkinson’s disease (PD), Parkinson’s disease with dementia (PDD), and dementia with lewy bodies (DLB) in brain regions with known increases in p-syn expression. Shown in A are westerns detecting SMG1 protein (α-SMG1) in the indicated cases. The control, PD, and PDD samples are from posterior cingulate cortex. Shown in B is a bar graph illustrating the differences in SMG1 expression between disease groups. Error bars are indicated on the graphs (n = 8 per group). Reductions in SMG1 expression in PD vs control and PDD vs either PD or control are statistically significant (p<0.05). Shown in C are SMG1 western results for control and DLB samples from temporal cortex. Shown in D is a bar graph quantifying the significant reduction in SMG1 expression in DLB compared to control temporal cortex.

While SMG1 knockdown clearly increases endogenous t-syn and p-syn levels in our cell line, it is difficult to gain insight into potential disease relevance using cell models. However, if reduced SMG1 expression contributes to pathological increases in t-syn or p-syn during the course of synucleinopathies, one would predict reduced expression of SMG1 protein in brain regions affected by p-syn neuropathology. To test this, we performed SMG1 western analyses on posterior cingulate cortex from patients that had either PD (n = 8) or PD with dementia (PDD, n = 8) (see Table 3) and compared SMG1 expression from these patients to healthy controls (n = 8) (Figure 7). The posterior cingulate cortex shows elevated p-syn levels in subjects showing Lewy-type synucleinopathy (LTS) at stage III of the Unified Staging System for Lewy Body Disorders [13], [27].

**Table 3:** Characteristics of patient samples.

Diagnosis (N)	Brain Region	Age	Gender(% Male)	PMI, hrs	No Lewy Bodies	Olfactory Bulb Onlyl (I)	BrainstemPredominant (IIa)	LimbicPredominant (Iib)	Brainstem & Limbic (III)	Neocortical (IV)
**Normal (8)**	PC	77.8 (7.0)	75	2.5 (0.7)	8 (100%)	0	0	0	0	0
**PD (8)**	PC	79.5 (7.3)	75	3.6 (3.3)	0	0	5 (63%)	2 (25%)	1 (12%)	0
**PDD (8)**	PC	75.7 (6.4)	67	2.8 (0.8)	0	0	0	0	6 (75%)	2 (25%)
**Normal (6)**	TC	80.1 (2.8)	50	2.7 (0.5)	6 (100%)	0	0	0	0	0
**DLB (6)**	TC	78.0 (4.4)	83	3.5 (0.6)	0	0	0	0	0	6 (100%)

General characteristics and Lewy Body staging of the study subjects, by neuropathologic diagnosis, brain region studied, age, gender, postmortem interval (PMI), and phosphorylated α-synuclein histopathology by the Unified Staging System for Lewy Body Disorders [27]. Means and standard deviations (SD) are given. Abbreviations: PD (Parkinson’s disease), PDD (Parkinson’s disease with dementia), DLB (dementia with Lewy bodies), PC (posterior cingulate cortex), TC (temporal cortex).

We found that SMG1 expression in PD was reduced to 46.2±25.1% (p = 0.021) of control levels (Figure 7). Surprisingly, SMG1 expression was not detected in the any of the eight PDD subjects tested. These results suggest that reduced SMG1 expression levels are associated with increases in p-syn during the progression of PD in this sample set. However, it will be important to expand this sample size in future work.

To determine if reduced SMG1 expression could be specific to PD and PDD, or might be more generalizable to other synucleinopathies, we assessed SMG1 expression in temporal cortex from 6 controls and 6 dementia with Lewy body (DLB) patients (see Table 3). DLB patients have elevated p-syn and overt Lewy pathology in the temporal cortex [13], [27]. In this sample set, SMG1 expression was significantly reduced in DLB patients to 31.7±4.2% (p = 0.0001) of control levels (Figure 7), suggesting a potentially more universal role for SMG1 in synucleinopathies beyond PD.

## Discussion

### Multiple Kinases and Phosphatases can Affect SNCA Levels *in vitro*

Published studies have thus far implicated numerous kinases in the phosphorylation of α-synuclein protein, including Polo-like Kinases (PLKs) 1, 2, and 3 [14], [15], [16], G-protein Coupled Receptor Kinases (GRKs) 1,2,5, and 6 [9], Leucine rich repeat kinase 2 (LRRK2) [28], and casein kinase 2 (CK2) [18]. Of these many kinases with *in vitro* pS129 phosphorylating activity, the PLKs have shown perhaps the most potential *in vivo* relevance thus far in animal models and in studies showing upregulation of some PLKs in the brains of individuals with synucleinopathies [14], [15]. These findings already suggested that the regulation of pS129 SNCA (p-syn) phosphorylation was likely to involve a complex signaling network of multiple kinases and phosphatases. Results from our comprehensive screen of 711 known or predicted kinases and 206 phosphatases affirm that conclusion. While we identified each of the PLKs as having a significant effect in our assay (Tables 1 and 2), we did not identify any of the other known kinases as potential regulators of p-syn. This may result from functional redundancies in the signaling pathways or from differences in the cell models used. Nevertheless, our results provide a starting point to further interrogate potential disease relevance of the kinases identified.

The role of phosphatases in the regulation of p-syn has been relatively understudied compared to the kinases. Some conflicting data surrounds a possible role for protein phosphatase 2A (PP2A) in dephosphorylating S129, with some studies supporting a role for this phosphatase [17] and some refuting a significant effect [29], [30]. We identified several subunits of PP2A (Table 2). However, siRNAs targeting these phosphatase subunits rather counterintuitively decreased the ratio of p-syn/t-syn. Additional studies will be needed to confirm that result and determine more precise mechanisms whereby decreases to this phosphatase could lead to decreases in p-syn. Only two phosphatases emerged from our screen at Z >2 with 2 siRNAs per target. These were dual specificity phosphatase 7 (DUSP7) and phosphatidylinositol-3-phosphate associated protein (PIP3AP). Further validation work will be required to determine any potential disease relevance of these phosphatases.

### SMG1 Affects α-syn Levels Independently of mRNA Surveillance

Knockdown of SMG1 expression using siRNA robustly increases expression of both total SNCA (t-syn) and and phospho-SNCA (p-syn) in our *in vitro* cell culture system (Figures 2 and 3). Moreover, these effects occur on the endogenously encoded α-synuclein and the α-synuclein produced from the Tet-off transgene. Given the widely known role of SMG1 in regulating mRNA surveillance pathways and the observation that t-syn levels, as opposed to only p-syn levels, were elevated, we sought to determine if SMG1 could be regulating α-synuclein protein expression through its mRNA surveillance activity. We found that knockdown of the UPF1 gene, which is the downstream target of SMG1 in the mRNA surveillance pathway and is required for mRNA surveillance activity, had no effect on p-syn or t-syn expression. This result suggests that mRNA surveillance mechanisms cannot explain SMG1’s effect on either t-syn or p-syn.

We similarly found that SMG1 does not signal through p53 to affect t-syn or p-syn levels. In contrast, knockdown of TIA1, a stress granule associated protein, partially mimicked the increased p-syn seen with SMG1 knockdown. We hypothesize that increased expression of α-synuclein may trigger a cellular stress response that results in the formation of stress granules to limit the production of α-synuclein protein. Under this model blockage of this response, either by knocking down SMG1 or TIA1, would remove this buffer to α-synuclein accumulation, resulting in significantly elevated t-syn and p-syn.

### Reduced SMG1 Expression in Pathologically Affected Brain Regions Suggests a Relevant Role in the Disease Process

Using a cell culture model, we have demonstrated directly that reducing SMG1 protein expression is sufficient to cause a marked and significant elevation of both t-syn and p-syn expression. We also provide evidence that SMG1 expression is significantly reduced in affected brain regions in at least two synucleinopathies (Figure 7). In the case of PD and PDD, the posterior cingulate cortex is a brain region of particular interest. Progression from PD to PDD is associated with mature Lewy body pathology and elevation of p-syn in this brain region. Interestingly, we were unable to detect any SMG1 expression in the posterior cingulate of PDD patients. We were able to detect a reduced level of SMG1 expression in PD patients, which do not have overt Lewy pathology in the posterior cingulate. Recent work by Lue *et al*. [13] indicates that p-syn levels are significantly elevated in the cingulate of Unified Lewy-type synucleinopathy (LTS) histopathology stage III PD patients prior to the onset of dementia, even though overt Lewy pathology is absent. Thus we conclude that, in PD and PDD patients, reduced SMG1 expression occurs in a brain region with elevated p-syn levels.

In dementia with Lewy bodies (DLB), significant Lewy pathology develops in temporal cortex. We found SMG1 expression to be significantly reduced in this brain region in DLB patients compared to cognitively normal controls that were free of Lewy-type histopathology (Figure 7). This result, in combination with results for PD and PDD, suggests the possibility that reduced SMG1 expression, which can clearly drive elevations to t-syn and p-syn *in vitro*, may be functionally relevant to the etiology of synucleinopathies *in vivo*, and suggests that further studies are warranted to delineate potential contributions of SMG1 function in synucleinopathies.

## Conclusions

Through a comprehensive siRNA screening against kinase and phosphatase, we identified phosphatidylinositol 3-kinase related kinase SMG1, significantly enhances expression of both p-Syn and t-Syn levels when knocked down. The SMG1 protein has known roles in multiple cellular functions, including DNA damage responses, telomere maintenance, mRNA surveillance, and a more recently described role in stress granule formation. We have ruled out known functions in mRNA surveillance (Figure 4) and p53-mediated responses (Figure 5). In addition, SMG1 dependent functions in dissociating telomeric repeat-containing RNA (TERRA) from telomeric foci are also likely not relevant here because this process requires UPF1 activity [31], [32], which is not involved in SMG1 effects on α-synuclein.

Knockdown of the cytotoxic granule-associated RNA binding protein (TIA1) showed a phenotypic response similar to SMG1 knockdown (Figure 6), albeit less dramatic in magnitude. This phenotype of TIA1 knockdown suggests that stress granule formation may be one cellular mechanism to reduce the accumulation of α-synuclein protein that may otherwise become cytotoxic. However, while SMG1 and TIA1 knockdown have similar phenotypes with respect to p-syn accumulation, and the lack of a synergistic effect of simultaneous knockdown of these two proteins suggests a common pathway, more work will be required to clarify if these two proteins exert their effects on α-synuclein through their known functions in stress granule formation. Reduced SMG1 expression in pathologically affected brain regions is consistent with a role for SMG1 in the generation of α-synuclein pathology. Whether SMG1 or the stress granule pathway may ultimately be exploitable for therapeutic benefit in synucleinopathies remains to be determined.

## Supporting Information

Figure S1 **Fluorescence detection indicating that SMG1 silencing increases p-syn and t-syn.** Shown is a 20× image of cells fluorescently labeled for nuclei (blue), t-syn (green), and p-syn (red). NS indicates non-silencing siRNA control. SMG1 siRNA are the panels in the right column. % NS control fluorescence intensity is indicated on the right of each row of images.
(TIF)

Table S1 **All siRNA screening data.** Shown in the table are the gene symbol, Entrez ID number, full gene name, siRNA library set from which siRNAs were drawn, the specific siRNA sequences for all siRNAs, the Z score for each siRNA, and the fold change in the ration of pSyn/tSyn relative to non-silencing siRNA control.
(XLSX)
