# Supplementary figures and images for: SMG1 Identified as a Regulator of Parkinson’s Disease-Associated alpha-Synuclein through siRNA Screening
Source: PLoS One. 2013 Oct 30;8(10):e77711. doi: 10.1371/journal.pone.0077711 (PMC3813773; doi:10.1371/journal.pone.0077711)

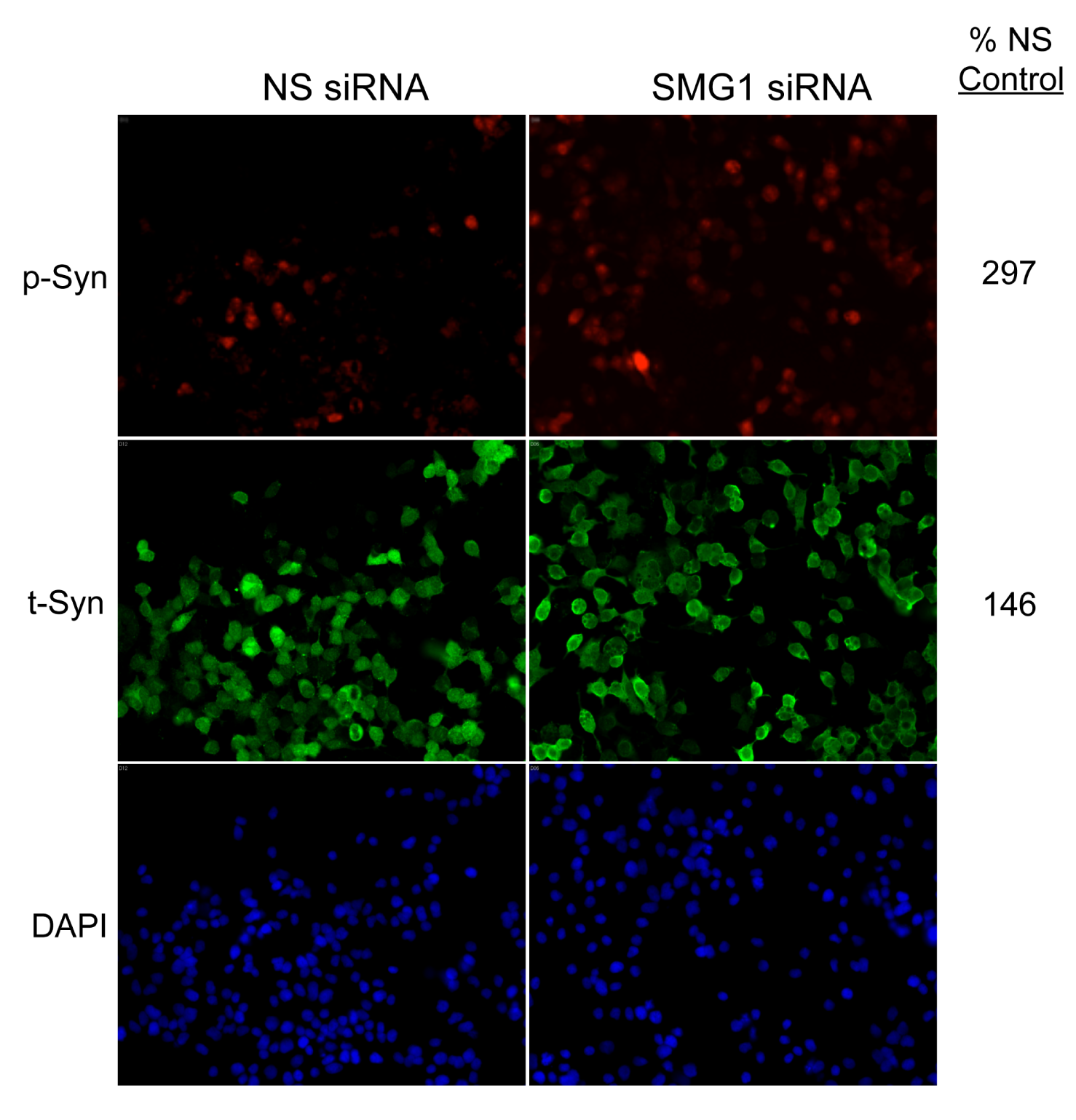

Supplement: Figure S1 — Fluorescence detection indicating that SMG1 silencing increases p-syn and t-syn. Shown is a 20× image of cells fluorescently labeled for nuclei (blue), t-syn (green), and p-syn (red). NS indicates non-silencing siRNA control. SMG1 siRNA are the panels in the right column. % NS control fluorescence intensity is indicated on the right of each row of images. (TIF) [file pone.0077711.s001.tif]
